# Genetic Susceptibility to Chronic Liver Disease in Individuals from Pakistan

**DOI:** 10.3390/ijms21103558

**Published:** 2020-05-18

**Authors:** Asad Mehmood Raja, Ester Ciociola, Imran Nazir Ahmad, Faisal Saud Dar, Syed Muhammad Saqlan Naqvi, Muhammad Moaeen-ud-Din, Ghazala Kaukab Raja, Stefano Romeo, Rosellina Margherita Mancina

**Affiliations:** 1 University Institute of Biochemistry and Biotechnology, Pir Mehr Ali Shah Arid Agriculture University Rawalpindi, Rawalpindi 46300, Pakistan; asadscm750@gmail.com (A.M.R.); saqlan@uaar.edu.pk (S.M.S.N.); ghazala@uaar.edu.pk (G.K.R.); 2 Department of Molecular and Clinical Medicine, The Sahlgrenska Academy at the University of Gothenburg, Wallenberg Laboratory, 413 45 Gothenburg, Sweden; ester.ciociola@wlab.gu.se; 3 Department of Pathology and Laboratory Medicine, Shifa International Hospitals Ltd., Islamabad 44790, Pakistan; imranazir@hotmail.com; 4 Liver Transplantation, Hepatobiliary and Pancreatic Services Unit, Shifa International Hospitals Ltd., Islamabad 44790, Pakistan; faisal.saud.dar@gmail.com; 5 Department of Animal Breeding and Genetics/National Center for Livestock Breeding, Genetics & Genomics, Pir Mehr Ali Shah Arid Agriculture University Rawalpindi, Rawalpindi 46300, Pakistan; m.moaeenuddin@uaar.edu.pk; 6 Department of Cardiology, Sahlgrenska University Hospital, 413 45 Gothenburg, Sweden; 7 Clinical Nutrition Unit, Department of Medical and Surgical Sciences, University Magna Graecia, 88100 Catanzaro, Italy

**Keywords:** NAFLD, Asia, ADPN, adiponutrin, TMC4, LPIAT1

## Abstract

Chronic liver disease, with viral or non-viral etiology, is endemic in many countries and is a growing burden in Asia. Among the Asian countries, Pakistan has the highest prevalence of chronic liver disease. Despite this, the genetic susceptibility to chronic liver disease in this country has not been investigated. We performed a comprehensive analysis of the most robustly associated common genetic variants influencing chronic liver disease in a cohort of individuals from Pakistan. A total of 587 subjects with chronic liver disease and 68 healthy control individuals were genotyped for the *HSD17B13* rs7261356, *MBOAT7* rs641738, *GCKR* rs1260326, *PNPLA3* rs738409, *TM6SF2* rs58542926 and *PPP1R3B* rs4841132 variants. The variants distribution between case and control group and their association with chronic liver disease were tested by chi-square and binary logistic analysis, respectively. We report for the first time that *HSD17B13* variant results in a 50% reduced risk for chronic liver disease; while *MBOAT7; GCKR* and *PNPLA3* variants increase this risk by more than 35% in Pakistani individuals. Our genetic analysis extends the protective role of the *HSD17B13* variant against chronic liver disease and disease risk conferred by the *MBOAT7*; *GCKR* and *PNPLA3* variants in the Pakistani population.

## 1. Introduction

Chronic liver disease, including viral and non-viral etiology, is endemic in many countries and is a growing burden for society and health-care systems. After the reduction of viral infections in the last 20 years in Western countries, non-viral liver disease is the most common chronic liver disease worldwide [1,2]. However, irrespective of the cause, the burden of this disease in many Middle Eastern and Asian countries is continuously rising [3]. Among Asian countries, with 9–10 million people having viral infection [3] and 10-fold more having non-viral liver disease [4], Pakistan represents the country with the highest prevalence of chronic liver disease.

Chronic liver disease has a strong genetic component [5,6]. Among all the genetic determinants of chronic liver disease, the *Patatin-like phospholipase domain-containing protein 3* (*PNPLA3*, also known as *Adiponutrin [ADPN]*) rs738409 (I148M) [7], *Membrane Bound O-Acyltransferase Domain Containing 7* (*MBOAT7*, also known as *Lysophosphatidylinositol-acyltransferase-1* [*LPIAT1*]) rs641738 [8,9,10], *Glucokinase regulator* (*GCKR)* rs1260326 (P446L) [11] and *Transmembrane 6 Superfamily Member 2* (*TM6SF2)* rs58542926 (E167K) [12,13] represent the most robust and widely validated genetic factors increasing the susceptibility to chronic liver disease. Recently, the *Hydroxysteroid 17-Beta Dehydrogenase 13* (*HSD17B13*) rs7261356 [14,15] and the *Protein Phosphatase 1 Regulatory Subunit 3B (PPP1R3B)* rs4841132 [16,17] have been shown to confer protection against liver disease, at least in at-risk individuals, instead. The proteins encoded by these genes are all involved in the hepatic lipid metabolism. More specifically: PNPLA3 is a lipase localized on the hepatic lipid droplets surface [18] with a triglyceride hydrolase activity (in hepatocytes) [19,20] and a retinyl-palmitate lipase activity (in hepatic stellate cells) [21]; MBOAT7 is a transmembrane acyltransferase protein localized on the endoplasmic reticulum, mitochondria-associated membranes and lipid droplets [8,22], involved in the hepatic phospholipids remodeling [23] and in a non-canonical hepatic triglyceride synthesis pathway fuelled by the phosphatidylinositol turnover [24]; GCKR is involved in the hepatic glucose metabolism [25] and responsible of inverse modulation of fasting plasma glucose and triglyceride levels [26,27]; TM6SF2 is involved in the APOB lipidation and modulates the hepatic lipid droplets secretion of triglycerides through very-low-density lipoproteins (VLDL) [13,28,29,30]; HSD17B13 is expressed on the hepatic lipid droplet membrane [14], is involved in the hepatic lipogenesis and modulates lipid droplets number and size [31]; PPP1R3B is primarily involved in the hepatic glycogen synthesis and, as results of this, secondarily modulates hepatic triglycerides synthesis through insulin-dependent de novo lipogenesis [16]. The fact that the main genetic determinants of liver disease encode for proteins involved in the hepatic lipid metabolism supports the role of dysregulated lipid metabolism in the pathogenesis of both viral and non-viral liver disease.

Despite the high prevalence of chronic liver disease in Pakistan, the genetic susceptibility to this condition in this country has not been investigated.

In this work, we performed a comprehensive analysis of the most robustly associated common genetic variants influencing chronic liver disease in a cohort of 655 Pakistani individuals comprising 587 individuals with chronic liver disease and 68 healthy controls. We showed for the first time a lower frequency of the minor and protective allele for *HSD17B13* variant and a higher frequency of the minor and risk allele for *MBOAT7* and *GCKR* in those with chronic disease. We also confirmed higher frequency of the risk allele for *PNPLA3* in individuals with chronic disease as compared to healthy controls. 

## 2. Results

A total of 655 Pakistani individuals comprising 587 individuals with chronic liver disease and 68 healthy controls were included in the present study (Table 1). Individuals from the two study groups were all adults with a mean BMI of 26 kg/m^2^ and a higher proportion of men. In the case group, the majority of the individuals were affected by viral chronic liver disease (89% viral vs. 11% with non-viral etiology), 34% had hepatocellular carcinoma (HCC) and 66% had cirrhosis. Additionally, compared to the control group, individuals from the case group had a higher proportion of men, higher ALT and AST, and lower total cholesterol, LDL, HDL and triglycerides.

### 2.1. The Frequency of Risk Alleles for Liver Disease Is Higher in Individuals with Chronic Liver Disease

To assess the genetic susceptibility to chronic liver disease in the Pakistani population, we compared the distribution of the minor allele frequencies of the variants between the case and control group. For these analyses, we included in the control group additional data on frequency distribution from 96 additional Pakistani individuals (Punjabi in Lahore) from 1000 Genomes Project (Ensembl release 99—January 2020).

**Figure 1:**
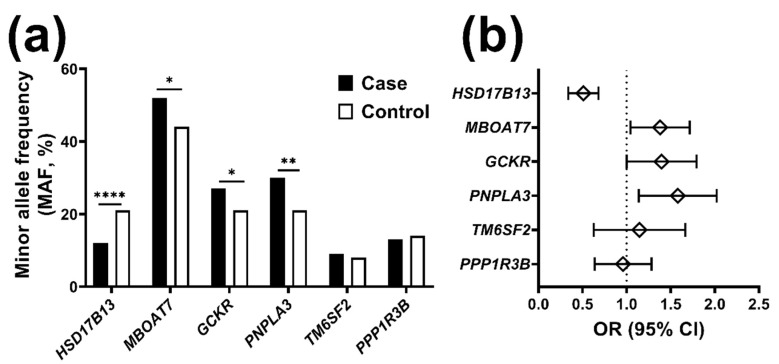
Minor allele frequency of main genetic determinants of liver disease and the corresponding risk for chronic liver disease in Pakistani individuals. (**a**) Minor allele frequency of main genetic determinants of liver disease stratified by case and control group. Data are shown as percentage. P-values calculated by chi-square. (**b**) The risk for chronic liver disease conferred by the minor allele of the main genetic determinants of chronic liver disease expressed as Odds ratio (OR) and its 95% confidence interval (CI). The risk was estimated by binary logistic regression under an additive genetic model with the exception of *TM6SF2* where a dominant genetic model has been assumed, considering the relative lower MAF. Case group: *n* = 587 individuals with chronic liver disease (*n* = 522 viral and 62 non-viral). Control group: *n* = 164 individuals without liver disease (*n* = 68 recruited in the present study plus 96 Punjabi individuals reported on 1000 Genomes Project ((Ensembl release 99—January 2020)). Abbreviations: *HSD17B13*, Hydroxysteroid 17-Beta Dehydrogenase 13; *MBOAT7*, Membrane Bound O-Acyltransferase Domain Containing 7; *PNPLA3*, Patatin-like phospholipase domain-containing protein 3; *CGKR*, Glucokinase regulator; *TM6SF2*, Transmembrane 6 Superfamily Member 2; *PPP1R3B*, Protein Phosphatase 1 Regulatory Subunit 3B. * *p* < 0.05; ** *p* < 0.005; *** *p* < 0.0001; **** *p* < 0.00005.

The frequency of the minor and protective allele for the *HSD17B13* variant was lower in individuals from the case group as compared to the control group (MAF = 0.12 vs. 0.21, respectively, *p*-value = 1.7 × 10^−5^, Figure 1a), suggesting a protective role of this allele against chronic liver disease. Additionally, the frequency of the minor and risk allele for *MBOAT7, GCKR,* and *PNPLA3* was higher compared to the control group (*p*-value = 0.017, 0.036 and 0.003 for the three variants, respectively), suggesting a chronic liver disease risk susceptibility conferred by these variants. We found no difference in the minor allele frequencies between individuals from the case and control group for *TM6SF2* nor for *PPP1R3B* variant. Results were similar when including only the 68 healthy control individuals recruited for the present study (data not shown).

### 2.2. The HSD17B13 Minor Allele Reduces the Risk for Chronic Liver Disease

To assess the risk for liver disease conferred by each variant, we performed univariate binary logistic regression analyses. We found that the *HSD17B13* variant was associated with an approximately 50% reduced risk for chronic liver disease (OR 0.49, CI 0.35–0.69, *p*-value = 2.6 × 10^−5^), *MBOAT7* and *GCKR* variants were associated with an approximately 35% increased risk for chronic liver disease (OR 1.35, CI: 1.06–1.73, *p*-value = 0.017, and OR 1.36, CI: 1.02–1.81, *p*-value = 0.036 respectively), and *PNPLA3* variant was associated with more than 50% increased risk for chronic liver disease (OR 1.54, CI: 1.162.04, *p*-value = 0.003) (Figure 1b).

## 3. Discussion

In the present study, we examined six common genetic variants robustly associated with chronic liver disease in individuals from Pakistan. We compared the distribution of the minor allele frequency of these variants between 587 individuals with and 164 individuals without chronic liver disease (*n* = 68 recruited in the present study plus 96 Punjabi individuals reported on 1000 Genomes Project (Ensembl release 99—January 2020)). As expected, and consistently with the presence of chronic liver disease, individuals from the case group had higher ALT and AST compared to individuals from the control group. Interestingly, individuals from the case group had lower total cholesterol, LDL, HDL and triglycerides compared to those from the control group. Our finding is in line with previously reported data showing that individuals with HCV or HBV-related cirrhosis have lower lipid profile (including total, LDL, and HDL cholesterol, and triglyceride) compared to healthy control individuals [32,33] and that serum lipid levels diminish linearly with the progression of liver damage [34].

We showed for the first time that *HSD17B13* variant is protective while *MBOAT7* and *GCKR* confer increased risk of chronic liver disease in Pakistani individuals.

Of note, for *HSD17B13* sequence variant conferred approximately 50% reduced risk for chronic liver disease in this cohort. *HSD17B13* rs7261356 is a splice loss-of-function variant resulting in the synthesis of a truncated protein with reduced expression levels [14]. It has been previously shown that this variant reduced the risk for liver disease in different ethnicities and regardless the etiology of liver disease. Indeed, the protective effect of the *HSD17B13* variant against non-alcoholic liver disease has already been shown in individuals of European [14], African [35] and Hispanics/Latinos descent [35,36]. The association between *HSD17B13* variant and reduced risk for advanced liver disease has also been shown in viral [37], alcoholic [38], portal hypertension [39], and in Wilson’s disease [40]. Despite the number of studies on *HSD17B13* and liver disease in American and European countries, to our knowledge there are no data available so far in Asian countries. 

The present study is the first extending the association between *HSD17B13* and protection against chronic liver disease to an Asian population, namely Pakistani. Our data are consistent with those reported for other ethnicities and, together with them, underline the potential of *HSD17B13* as a therapeutic target [41] to treat liver disease irrespective of ethnicity, and its role in the genetic susceptibility to chronic liver disease.

With the present study, we also show for the first time that the minor and risk allele for the *MBOAT7* rs641738 and for *GCKR* rs1260326 variants are enriched in Pakistani individuals with chronic liver disease and each of them induces a 35% increased risk for chronic liver disease. The *MBOAT7* variant, originally reported as intergenic variant between *MBOAT7* and *Transmembrane Channel Like 4* (*TMC4*), is located in the 3’UTR of the *MBOAT7* gene and results in its decreased expression level, which consequently induces liver disease by altering phosphatidylinositol remodeling [8] and by triggering a novel non-canonical hepatic triglyceride synthesis pathway fuelled by phospholipid turnover [24]. Our findings are in line with results previously reported in other ethnicities describing the association between the *MBOAT7* variant and chronic liver disease and suggest that the rs641738 is the causal genetic variant influencing expression level of MBOAT7 and increasing the risk to liver disease [8,9].

The *GCKR* rs1260326 is a missense variant encoding for a proline (P) to leucine (L) at position 446 of the protein (P446L). The minor allele (L) for this variant has been previously associated with higher prevalence of liver disease and lower prevalence of type-2 diabetes or lower levels of glycaemic traits [11]. Our findings confirm the role of the *GCKR* variant in the genetic susceptibility to liver disease. However, glycaemic traits were not available for our study groups.

The *PNPLA3* rs738409 is a missense variant encoding for an isoleucine to methionine substitution at position 148 of the protein (I148M). It increases the susceptibility to liver disease by affecting both triglyceride [18,42], and retinol remodeling in the liver [21,43], and increasing the production of profibrotic proteins from hepatic stellate cells [44,45]. Very recently, it has been highlighted that inhibition of the mutant *Pnpla3* may be used as a therapeutic target to treat fatty liver disease [46,47]. This variant represents the first and the most widely validated genetic determinant of liver disease [7]. Its role in liver disease onset and progression has been already demonstrated in different ethnicities, including some from Asia [48,49,50,51,52,53]. We found that the frequency of *PNPLA3* minor allele was higher in individuals with chronic liver disease than in healthy controls and that each minor allele was associated with more than 50% increased risk for chronic liver disease. Our findings are in line with previously reported data from both European and Asian populations and, for the first time, extend the association between *PNPLA3* variant and chronic liver disease in a Pakistani population.

We additionally tested the effect of *TM6SF2* rs58542926 and *PPP1R3B* rs4841132 variant on the genetic susceptibility to chronic liver disease in the Pakistani population. The *TM6SF2* rs58542926 encodes for a glutamic acid to lysine amino acidic substitution at position 167 of the protein (E167K). This variant increases the risk for chronic liver disease by affecting the secretion of APO-B-containing lipoproteins [12,13]. On the other hands, the *PPP1R3B* rs4841132 is located 175 kilobase (kb) upstream of the *PPP1R3B* coding region and is associated with reduced susceptibility to chronic liver disease in at risk individuals [6,16,17].

In our study, we did not detect any significant difference in the distribution of these two variants between individuals from the case and the control group although the trend was in the expected direction. Indeed, we found that the MAF for the *TM6SF2* variant was slightly higher while the MAF for the *PPP1R3B* variant was slightly lower in the case group compared to healthy individuals. This result is probably due to a lack of statistical power.

The tested variants are widely recognized as the main genetic determinants of liver disease. Of note, all the tested genes encode for proteins involved in the hepatic lipid metabolism. This supports the role of dysregulated lipid metabolism in the pathogenesis of both non-viral and viral liver disease. Of note, in HCV-related liver disease, there is a growing body of evidence linking the HCV life cycle and hepatocytes’ lipid metabolism [54]. More specifically, it has been shown that the HCV core accumulates around the hepatocytes’ lipid droplets (storage of triglycerides) [55] and inhibits the secretion of VLDL [56], the APOB-containing triglycerides-rich lipoproteins. In the lipid droplets, HCV virus particles are packed with APOB and then secreted as a lipo-viral particle (LVP) in the blood circulation. Then, these particles may enter again into hepatocytes through the LDL-receptor [57], the surface receptor that is responsible for the hepatic APOB-mediated LDL-cholesterol uptake. All this together suggests that APOB is deeply involved in the HCV life cycle [58]. Many studies investigated the genetic association between *APOB* and HCV-related viral disease showing that variants in *APOB* increase the susceptibility to HCV infection [59,60]. So far, variants on *APOB* were mainly investigated for associations with cardiovascular disease showing a different effect based on population ethnicity [61]. It would be important to investigate if this could be the case for the susceptibility to HCV as well. Additionally, it would be important to test whether APOB polymorphisms may modulate the risk or the protection of chronic liver disease conferred by variants on *HSD17B13*, *MBOAT7*, *PNPLA3*, or *CGKR*.

The relatively low sample size together with the absence of glycemic traits and the use of a combined cohort with different etiologies (viral and non-viral), represent the main limitation of this study. Additionally, the lack of clinical and anthropometric data for individuals from the 1000 Genome project, does not allow to perform multivariate analysis to evaluate the contribution of each tested minor allele on the risk for liver disease independently from confounders. The major strength of this study, on the other hand, is represented by the number of common variants tested, which provide comprehensive analyses of the genetic susceptibility to chronic liver disease in the Pakistani population.

In conclusion, our genetic analysis of common variants related to chronic liver disease extends the protective role of the *HSD17B13* variant against chronic liver disease and the increased susceptibility risk to this disease conferred by the presence of the *MBOAT7* rs641738, *GCKR* rs1260326 or *PNPLA3* rs738409 variant in a Pakistani population. Considering the potential lack of power of our study to detect differences in the *TM6SF2* rs58542926 and *PPP1R3B* rs4841132 frequency between the two study groups, larger studies are recommended to test the contribution of these variants on chronic liver disease in Pakistani population.

## 4. Materials and Methods 

### 4.1. Study Cohort

For the case group: a total of 587 consecutive Pakistani individuals with chronic liver disease (*n* = 522 viral and 62 non-viral), who underwent living donor liver transplantation (LDLT) for end stage liver disease, were recruited from April 2012 to July 2018 at the Liver Transplantation, Hepatobiliary and Pancreatic Services unit, Shifa International Hospitals Ltd. (Islamabad, Pakistan). All causes of cirrhosis or hepatocellular carcinoma with the exception for fulminant hepatic failure were considered eligible for the present study. Diagnosis of liver disease was performed based on clinical history, biochemical parameters and histopathology reports.

For the control group: a total of 68 healthy individuals without liver disease of any etiology and without metabolic syndrome, defined as a combination of complex risk factors including central obesity (BMI > 29.9 kg/m^2^), hypertension (SBP ≥ 130mmHg/DBP ≥ 85 mmHg), dysglycemia (fasting blood glucose >100 mg/dL), and dyslipidemia (triglycerides > 200 mg/dL and HDL < 40 mg/dL) [62], were randomly recruited from general Pakistani population. To this group, for the minor allele frequency comparisons between case and control group, genotypes information from 96 Punjabi individuals reported on 1000 Genomes Project (Ensembl release 99—January 2020)) were included. The 1000 Genome project is a selection-bias-free population-based collection of reference genomic sequences aiming to provide a comprehensive description of common human genetic variations of individuals from multiple populations [63]. Thus, individuals from the 1000 Genome project represents a correct setting to evaluate if there is an enrichment of a target risk variant in a selected study group compared to the general population. However, information on phenotype or medical conditions of the study participants are held in confidence by the local investigators and not publicly available.

Information about age, gender and BMI were registered at the time of enrollment for all the recruited study participants. Measurement of clinical parameters including lipid profile (total Cholesterol, LDL, HDL and Triglyceride), transaminases (ALT and AST), and viral serology (Hepatitis B surface antigen, Hepatitis B core antibody, Hepatitis C virus antibody and Hepatitis Delta virus antibody) were performed on freshly isolated blood samples and under starvation conditions at the time of transplantation (case group) or at the time of enrollment (control group).

Formalin Fixed Paraffin Embedded (FFPE) blocks of explant liver for DNA extraction were collected from the pathology laboratory at the time of transplantation for individuals from the case group.

Extra EDTA-containing blood tubes for DNA extraction were collected at the time of the enrollment for the individuals from the control group.

Informed written consent was obtained from each subject, and the study protocol was approved by the PMAS Arid Agriculture University Rawalpindi’s (PMAS AAUR) Ethics Committee for use of Human Subjects and Institutional Review Board and Ethics Committee (IRB & EC) of Shifa International Hospitals Ltd. (Islamabad) IRB# 834-109-2017.

### 4.2. DNA Extraction and Genotyping

DNA was extracted from Formalin-Fixed Paraffin-Embedded (FFPE) liver specimens using RecoverAll^TM^ total nucleic acid isolation kit for FFPE (Invitrogen^TM^, ThermoFisher Scientific, USA) according to manufactures’ instructions (case group), or from EDTA-containing blood by the salting-out method [64] (control group).

*HSD17B13* rs7261356, *MBOAT7* rs641738, *GCKR* rs1260326, *PNPLA3* rs738409, *TM6SF2* rs58542926, and the *PPP1R3B* rs4841132 variants were genotyped in duplicate (with a 100% concordance between duplicate) by TaqMan 5’-nuclease assays (Thermo Fisher Scientific, Waltham, MA, USA). Assays were already available and directly provided by Thermo Fisher Scientific (TaqMan) for all of the target genetic variants except for *HSD17B13* rs7261356 for which a custom assay was designed as follows: Context sequence: TATTTGGGTGTTCTGTGCTGTACTT[*/A]ACTTCTGTAGTCTCAGAAAGATATT; forward primer: GCTCTATTGGTGTTTTAGTATTTGGGTGTT; reverse primer: GAAGTCTGATAGATGGAATACTTACCAATAAGA. Post-PCR allelic discrimination was performed by measuring allele-specific fluorescence using a CFX384 Real-Time System (Bio-Rad Laboratories Inc, Hercules, CA, USA). In the subset of healthy control individuals, Hardy Weinberg equilibrium was preserved for all the variants. In the subset of the individuals with chronic liver disease, Hardy Weinberg equilibrium was deviated for *PNPLA3* rs738409, *GCKR* rs1260326, and *PPP1R3B* rs4841132 probably due to the bias of selection.

### 4.3. Statistical Analysis

For descriptive statistics, continuous traits are shown as mean and standard deviation (normally distributed traits) or as median and quartile range (non-normally distributed traits), gender is shown as number and proportion. P-values for comparison of continuous traits between case and control group were calculated by linear regression analysis adjusted for age, gender and BMI when appropriate. Non-normally distributed traits have been log-transformed before entering the model. P-value for gender distribution was calculated by Chi-square. After Bonferroni multiple testing correction, only *p* < 0.005 (5.0 × 10^−3^) was considered significant.

Data for minor allele frequency (MAF) are shown as percentage. Data for the risk for liver disease conferred by the minor allele of the main genetic determinants of chronic liver disease are expressed as Odds ratio (OR) and its 95% confidence interval (CI). P-values for comparison of MAF between case and control group have been calculated by Chi-square. The risk for chronic liver disease has been evaluated by binary logistic analysis under an additive genetic model (except for *TM6SF2* variant where a dominant genetic model has been assumed considering the relatively low MAF).

Statistical analyses were carried out using the IBM Statistical Package for Social Sciences (IBM SPSS, version 26, Inc. Chicago, IL, USA). *p*-values < 0.05 were considered statistically significant.

## Figures and Tables

**Table 1:** Clinical characteristics of the two study groups.

Characteristic	Case	Control	*p-*Value
N	587	68	
Age, Years	48 ± 11	46 ± 12	0.166
Male gender, n (%)	476 (81)	42 (62)	2.1 × 10^−4^
BMI, Kg/m^2^	26 ± 5	26 ± 4	0.290
Cholesterol, mg/dL	88 ± 40	167 ± 35	2.3 × 10^−45^
LDL, mg/dL	51 ± 32	96 ± 41	1.9 × 10^−23^
HDL, mg/dL	19 ± 12	40 ± 9	1.2 × 10^−44^
Triglyceride, mg/dL	69 (54–100)	154 (120–191)	1.7 × 10^−25^
ALT, IU/L	66 (54–84)	28 (22–34)	7.1 × 10^−73^
AST, IU/L	61 (47–78)	29 (23–32)	2.7 × 10^−64^
NASH/Viral disease, n (%)	62/522 (11/89)	-	-
HCC/Cirrhosis, n (%)	197/390 (34/66)	-	-

For continuous traits, data are shown as mean and standard deviation (normally distributed traits) or as median and quartile range (non-normally distributed traits). Gender is shown as number and proportion. P-values for continuous traits were calculated by linear regression analysis adjusted for age, gender and BMI when appropriate. *p*-value for gender distribution was calculated by Chi-square. After Bonferroni multiple testing correction, only *p* < 0.005 (5.0 × 10^−3^) was considered significant.

## Abbreviations

ALT	Alanine transaminase
AST	Aspartate transaminase
BMI	Body mass index
CGKR	Glucokinase regulator
CI	Confidence interval
FFPE	Formalin-Fixed Paraffin-Embedded
HCC	Hepatocellular carcinoma
HDL	High-density lipoproteins
HSD17B13	Hydroxysteroid 17-Beta Dehydrogenase 13
LDL	Low-density lipoproteins
MBOAT7	Membrane Bound O-Acyltransferase Domain Containing 7
OR	Odds ratio.
PNPLA3	Patatin-like phospholipase domain-containing protein 3
PPP1R3B	Protein Phosphatase 1 Regulatory Subunit 3B
TM6SF2	Transmembrane 6 Superfamily Member 2
